# Neuropsychiatric adverse drug reactions with oral tyrosine kinase inhibitors in metastatic colorectal cancer: an analysis from the FDA Adverse Event Reporting System

**DOI:** 10.3389/fonc.2023.1268672

**Published:** 2023-10-31

**Authors:** Maria Antonietta Barbieri, Giulia Russo, Emanuela Elisa Sorbara, Giuseppe Cicala, Tindara Franchina, Mariacarmela Santarpia, Desirèe Speranza, Edoardo Spina, Nicola Silvestris

**Affiliations:** ^1^ Department of Clinical and Experimental Medicine, University of Messina, Messina, Italy; ^2^ Department of Human Pathology in Adulthood and Childhood Gaetano Barresi, University of Messina, Messina, Italy

**Keywords:** adverse drug reactions, colorectal cancer, encorafenib, neuropsychiatric disorders, oral tyrosine kinase inhibitors, regorafenib

## Abstract

**Introduction:**

New oral tyrosine kinase inhibitors (TKIs) are approved for metastatic colorectal cancer (mCRC). The aim of this study was to assess the neuropsychiatric adverse drug reactions (ADRs) of these drugs reported in the FDA Adverse Event Reporting System (FAERS) database.

**Methods:**

All reports with regorafenib (REG) and encorafenib (ENC) as the primary suspect, and reported in the FAERS between 2012 and 2022, were collected. A descriptive and disproportionality analyses were conducted.

**Results:**

Out of 4,984 cases, 1,357 (30.2%) reported at least one neuropsychiatric ADR. New potential signals for REG included neuropathy peripheral (*n* = 265; reporting odds ratio, ROR = 19.48, 95% confidence interval, CI 95% = 17.52-22.47; information component, IC = 2.89, IC_025_-IC_075 _= 2.77-3.02), hyperesthesia (*n* = 18; ROR = 12.56, CI 95% = 7.90-19.96; IC = 2.25, IC_025_-IC_075 _= 1.79-2.72), taste disorder (*n* = 41; ROR = 9.91, CI 95% = 7.29-13.49; IC = 2.18, IC_025_-IC_075 _= 1.88-2.49), poor quality sleep (*n* = 18; ROR = 6.56, CI 95% = 4.13-10.42; IC = 1.74, IC_025_-IC_075 _= 1.27-2.20), altered state of consciousness (*n* = 15; ROR = 5.50, CI 95% = 3.31-9.14; IC = 1.57, IC_025_-IC_075 _= 1.06-2.07), depressed mood (*n* = 13; ROR = 1.85, CI 95% = 1.07-3.19; IC = 0.58, IC_025_-IC_075 _= 0.04-1.13) and insomnia (*n* = 63; ROR = 1.48, CI 95% = 1.15-1.89; IC = 0.38, IC_025_-IC_075 _= 0.13-0.63). For ENC comprised depressed mood (*n* = 4; ROR = 5.75, CI 95% = 2.15-15.39; IC = 1.74, IC_025_-IC_075 _= 0.76-2.73) and cognitive disorders (*n* = 3; ROR = 4.71, CI 95% = 1.51-14.66; IC = 1.54, IC_025_-IC_075 _= 0.41-2.68).

**Discussion:**

This study identified new unknown potential neuropsychiatric ADRs. Further investigations are required to better define the neurotoxicity of TKIs in mCRC patients.

## Introduction

Colorectal Cancer (CRC) is characterized by specific molecular and mutational alterations that play an important role in the choice of treatment. Approximately 40% of CRC patients have KRAS mutations, while about 6% have NRAS mutations leading to the constitutive activation of Ras-Raf-mitogen-activated protein kinase (MAPK) signaling pathway, downstream of the epidermal growth factor receptor (EGFR) (1, 2). As a result, many CRC patients are resistant to anti-EGFR therapies (3, 4).

Other relevant genomic alterations include mutations in the BRAF gene, which encodes a serine/threonine kinase within the MAPK signaling pathway. Approximately 8-12% of CRC patients have a BRAF mutation and 95% of BRAF mutations result in a substitution of the amino acid valine with glutamic acid at position 600 (BRAFV600E) (5–7). Patients with BRAF mutations generally have a poor prognosis in CRC due to their resistance to conventional therapies. Oral BRAF inhibitors (BRAFi) have had a significant impact on the treatment approach and clinical outcomes for patients with metastatic colorectal cancer (mCRC) who are not considered suitable candidates or have been unsuccessfully treated with standard therapies (8, 9).

Regorafenib (REG), an oral multikinase inhibitor, since it inhibits antigenic and oncogenic kinases, such as vascular endothelial growth factor receptors (VEGFR), platelet-derived growth factor receptors (PDGFR), fibroblast growth factor receptors (FGFR), and BRAF, received approval from the US Food and Drug Administration (FDA) in September 2012 for the treatment of mCRC (10, 11). In 2020, a pure oral BRAFi encorafenib (ENC) was approved in combination with cetuximab (CET) (12). Oral tyrosine kinase inhibitors (TKIs) offer a better prognosis in terms of progression free survival and overall survival than conventional therapies, with several advantages over injectable formulations, such as flexibility, convenience, cost-effectiveness, and better compliance (13, 14).

Despite the positive impact on patients’ survival in mCRC, the utilization of REG and ENC is not exempt from the occurrence of adverse events (AEs): 91% of patients treated with REG experienced AEs, including fatigue (46%), hand-foot skin reaction (HFSR) (42%), hypertension (30%), diarrhea (25%), and oral mucositis (25%) (15). Gastrointestinal disorders such as diarrhea (38%), nausea (38%), and decreased appetite (31%) were the most commonly reported AEs for ENC, along with fatigue (33%), dermatitis acneiform (30%), asthenia (24%), arthralgia (23%), and headache (20%). Moreover, serious AEs (SAEs) such as anemia (6%) and intestinal obstruction (5%) were primarily reported (5). The safety profile of oral TKIs is not fully understood, especially concerning neurological and psychiatric AEs and the consequent worsening of patients’ quality of life (QoL). A post-marketing study found an increased reporting of cerebral infarction, including ischemic stroke, for REG (16). A possible role in the pathophysiology of neurological disorders of oral kinase inhibitors cannot be ruled out. Other pharmacovigilance studies described a possible occurrence of peripheral neuropathies in patients under treatment with BRAFi and/or MEK inhibitors (MEKi) for melanoma (17, 18). However, no safety studies based on the spontaneous reporting system (SRS) databases aim to evaluate REG- and ENC-related neuropsychiatric ADRs in mCRC.

For all these reasons, the objective of this study was to assess and describe all ADRs associated with oral TKIs in the treatment of mCRC, focusing on neuropsychiatric ADRs, by conducting an analysis of the US Food and Drug Administration’s Adverse Event Reporting System (FAERS) database.

## Materials and methods

### Data source and case definition

An observational study was conducted using reports from the FAERS database. The FAERS database contains more than 20 million reports of suspected ADRs gathered from United States, Europe, and Asia, reported by patients, healthcare professionals, or pharmaceutical companies. Each report includes an identification number, patient information (e.g., gender, age, and weight), reporting date, reporting country, qualifications of the primary reporter, suspected and concomitant drugs with their respective indications, date of onset of ADRs, seriousness, and description of each ADR coded by Preferred Term (PT) from the Medical Dictionary for Regulatory Activities (MedDRA^®^ 26.0), grouped by System Organ Class (SOC).

Each ADR was categorized as “serious”, in accordance with the International Council on Harmonization E2D guidelines, when it led to any of the following consequences: death; life-threatening; hospitalization or prolonged hospitalization; a persistent or significant incapacity or substantial disruption of the ability to conduct normal life functions; a congenital anomaly/birth defect; an important medical event (IME).

On January 30^th^ 2023, zipped ASCII files (from Q4 2012 to Q4 2022) were downloaded from the “FDA Adverse Event Reporting System (FAERS) Quarterly Data Extract Files” website (https://fis.fda.gov/extensions/FPD-QDE-FAERS/FPD-QDE-FAERS.html) and processed to eliminate duplicates, specifically those with overlapping information in key fields of AE, event date, gender, age, body weight, reporting country, and primary suspected active substances.

After reviewing all reports where the primary suspect was REG or ENC (ENC in association with CET as secondary suspect) and considering the indication of CRC in accordance with the FDA Prescribing Information, reports with at least one ADR related to the SOCs “Nervous system disorders” and “Psychiatric disorders” were selected and defined as “cases”. All other reports, that did not contain neuropsychiatric ADRs were considered as the reference group (“non-cases”). The selection process was conducted at the case level using the identification number. Reports with multiple neuropsychiatric ADRs were counted only once.

### Data analyses

The demographic and clinical characteristics were assessed in terms of gender and age group, reporter type, reporter country, year of reporting, seriousness, and outcome of the ADRs between cases and non-cases. For all neuropsychiatric ADRs, the time to onset (TTO) was calculated and expressed in days (median, interquartile range, Q1-Q3) as the difference from the start of the primary suspect drug to the date of onset of the ADR, when both were available. Categorical variables were reported as absolute and percentage values, and differences were estimated using the Pearson’s chi-square test with Yates’ continuity correction. Continuous variables were expressed as median (Q1-Q3), and differences were assessed using the Mann-Whitney U test.

Moreover, a disproportionality analysis was performed using a case/non-case methodology. The Reporting Odds Ratio (ROR), with the corresponding 95% confidence interval (CI), was calculated to identify potential reporting disproportionality signals in neuropsychiatric ADRs related to REG or ENC. An exploratory disproportionality approach comparing ADRs related to ENC or REG versus all other drugs (non-cases) reported in the FAERS database was conducted (19). All neuropsychiatric ADRs with a significant ROR (lower limit of the 95% CI >1 with at least 3 cases) (20) were carefully considered unexpected if they were not reported in the FDA label at the time of the study. The Bayesian Information Component (IC), estimated as significant by the 95% credibility interval > 0 (IC_025_> 0), which is more accurate with a low number of reports, was calculated to decrease the risk of detecting false signals (21, 22).

The Statistical Package for the Social Science (SPSS) version 23.0 software for Windows (IBM Corp. SPSS Statistics) was used to conduct all statistical analyses.

## Results

### Characteristics of reports

**Figure 1:**
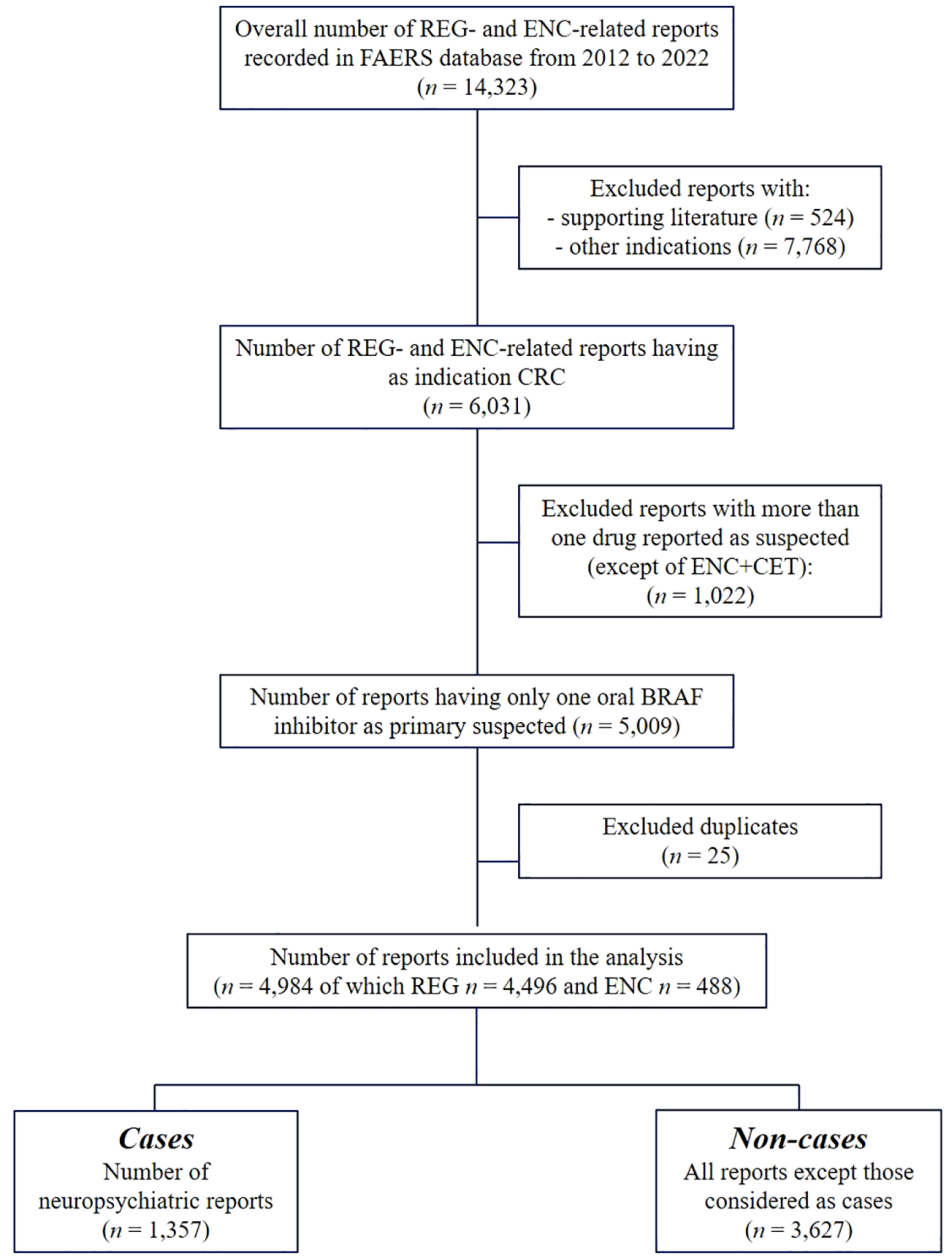
Flowchart of reports selection process. FAERS, US Food and Drug Administration’s Adverse Event Reporting System (FAERS) database; mCRC, metastatic colorectal cancer; REG, regorafenib; ENC, encorafenib; CET, cetuximab.

A total of 14,323 reports with REG or ENC as the suspect drug were collected in the FAERS database from October 2012 to December 2022. Premarketing reports with supporting literature (*n* = 524) and those related to other indications (*n* = 7,768) were not included in the analysis, as well as reports with another drug reported as the primary suspected (*n* = 1,022). Additionally, duplicates (*n* = 25) were excluded. Finally, 4,984 reports were included in the analysis, mainly related to REG (*n* = 4,496; 90.2%), followed by ENC (*n* = 488; 9.8%). Among the reports where ENC was identified as the primary suspect, 150 cases (30.7%) also implicated CET as a secondary suspected drug. Reports containing at least one neuropsychiatric ADR (cases) accounted for more than 1/4 of all reports (*n* = 1,357; 30.2%) (**Figure 1**).

The reports were almost equally distributed by gender, with no statistically significant difference between cases and non-cases. However, neuropsychiatric ADRs were more commonly related to adults compared to all other reports (49.5% *vs.* 41.1%, *p* = 0.003), particularly for ages 50 to 64 years (*n* = 547; 40.3%). Neuropsychiatric ADRs were mostly reported by consumers compared to other reports (69.5% *vs.* 32.6%, *p* < 0.001), during the years 2016 and 2021 (8.9% *vs*. 6.5%, *p* = 0.004 and 12.6% *vs.* 10.4%, *p* = 0.033, respectively), with North America being the main reporter country (67.6% *vs.* 40.2%, *p* < 0.001). Overall, cases showed a higher seriousness than non-cases (95.4% *vs.* 88.2%, *p* < 0.001), with a higher proportion of disabled and other outcomes (2.0% *vs.* 0.9%, *p* = 0.002 and 48.3% *vs.* 32.9%, *p* < 0.001, respectively). Neuropsychiatric ADRs mostly involved REG as the primary suspect compared to other reports (94.1% *vs.* 88.5%, *p* < 0.001) (**Table 1**).

**Table 1:** Characteristics of neuropsychiatric reports related to regorafenib or encorafenib.

Characteristic	Neuropsychiatric cases (*n* = 1,357)	Other reports (*n* = 3,627)	*P* value	Total (*n* = 4,984)
Gender, *n* (%)				
Male	723 (53.3)	1,891 (52.1)	0.488	2,614 (52.4)
Female	610 (45.0)	1,522 (42.0)		2,132 (42.8)
Not specified	24 (1.8)	214 (5.9)		238 (4.8)
Median age (Q1-Q3), years	64 (56–71)	64 (56-71)	0.530	64 (56-71)
Age group, *n* (%)				
Adult	672 (49.5)	1,488 (41.1)	0.003*	2,160 (43.3)
18-29 years	1 (0.1)	25 (0.7)	0.003	26 (0.5)
30-49 years	124 (9.1)	315 (8.7)		439 (8.8)
50-64 years	547 (40.3)	1,148 (31.7)		1,695 (34.0)
Elderly	531 (41.8)	1,451 (39.5)		2000 (40.1)
65-75 years	395 (29.1)	1,013 (27.9)	0.600	1,408 (28.3)
76-85 years	157 (11.6)	372 (10.3)		529 (10.6)
>85 years	15 (1.1)	48 (1.3)		63 (1.3)
Missing	118 (8.7)	706 (19.5)		824 (16.5)
Reporter type, *n* (%)				
Consumer	943 (69.5)	1,182 (32.6)	<0.001	2,125 (42.6)
Healthcare professional	413 (30.4)	2,398 (66.1)		2,811 (56.4)
Not specified	1 (0.1)	47 (1.3)		48 (1.0)
Reporter Country, *n* (%)				
Africa	3 (0.2)	32 (0.9)	0.022	35 (0.7)
Asia	168 (12.4)	993 (27.4)	<0.001	1,161 (23.3)
Europe	176 (13.0)	682 (18.8)	<0.001	858 (17.2)
North America	918 (67.6)	1,458 (40.2)	<0.001	2,376 (47.7)
Oceania	17 (1.3)	36 (1.0)	0.521	53 (1.1)
South America	31 (2.3)	122 (3.4)	0.061	153 (3.1)
Not specified	44 (3.2)	304 (8.4)	–	348 (7.0)
Serious, *n* (%)	1,294 (95.4)	3,198 (88.2)	<0.001	4,492 (90.1)
Outcome, *n* (%)				
Died	188 (13.9)	860 (23.7)	<0.001	1,048 (21.0)
Disabled	27 (2.0)	31 (0.9)	0.002	58 (1.2)
Hospitalized	407 (30.0)	1,016 (28.0)	0.179	1,423 (28.6)
Life threatening	16 (1.2)	95 (2.6)	0.003	111 (2.2)
Non-serious	63 (4.6)	429 (11.8)	<0.001	492 (9.9)
Other outcomes	655 (48.3)	1,194 (32.9)	<0.001	1,849 (37.1)
Required intervention	1 (0.1)	2 (0.1)	0.812	3 (0.1)
Year of reporting, *n* (%)				
2012	24 (1.8)	57 (1.6)	0.716	81 (1.6)
2013	149 (11.0)	510 (14.1)	0.005	659 (13.2)
2014	111 (8.2)	399 (11.0)	0.004	510 (10.2)
2015	151 (11.1)	387 (10.7)	0.680	538 (10.8)
2016	121 (8.9)	236 (6.5)	0.004	537 (7.2)
2017	131 (9.7)	344 (9.5)	0.899	475 (9.5)
2018	126 (9.3)	340 (9.4)	0.967	466 (9.3)
2019	108 (8.0)	292 (8.1)	0.962	400 (8.0)
2020	150 (11.1)	333 (9.2)	0.053	483 (9.7)
2021	171 (12.6)	378 (10.4)	0.033	549 (11.0)
2022	115 (8.5)	351 (9.7)	0.214	466 (9.3)
Primary suspect drug				
ENC	72 (5.3)	416 (11.5)	<0.001	488 (9.8)
REG	1,285 (94.7)	3,211 (88.5)		4,496 (90.2)

ENC, encorafenib; Q1, quartile 1; Q3, quartile 3; REG, regorafenib.

*Calculated Adults vs. Elderly.

**Figure 2:**
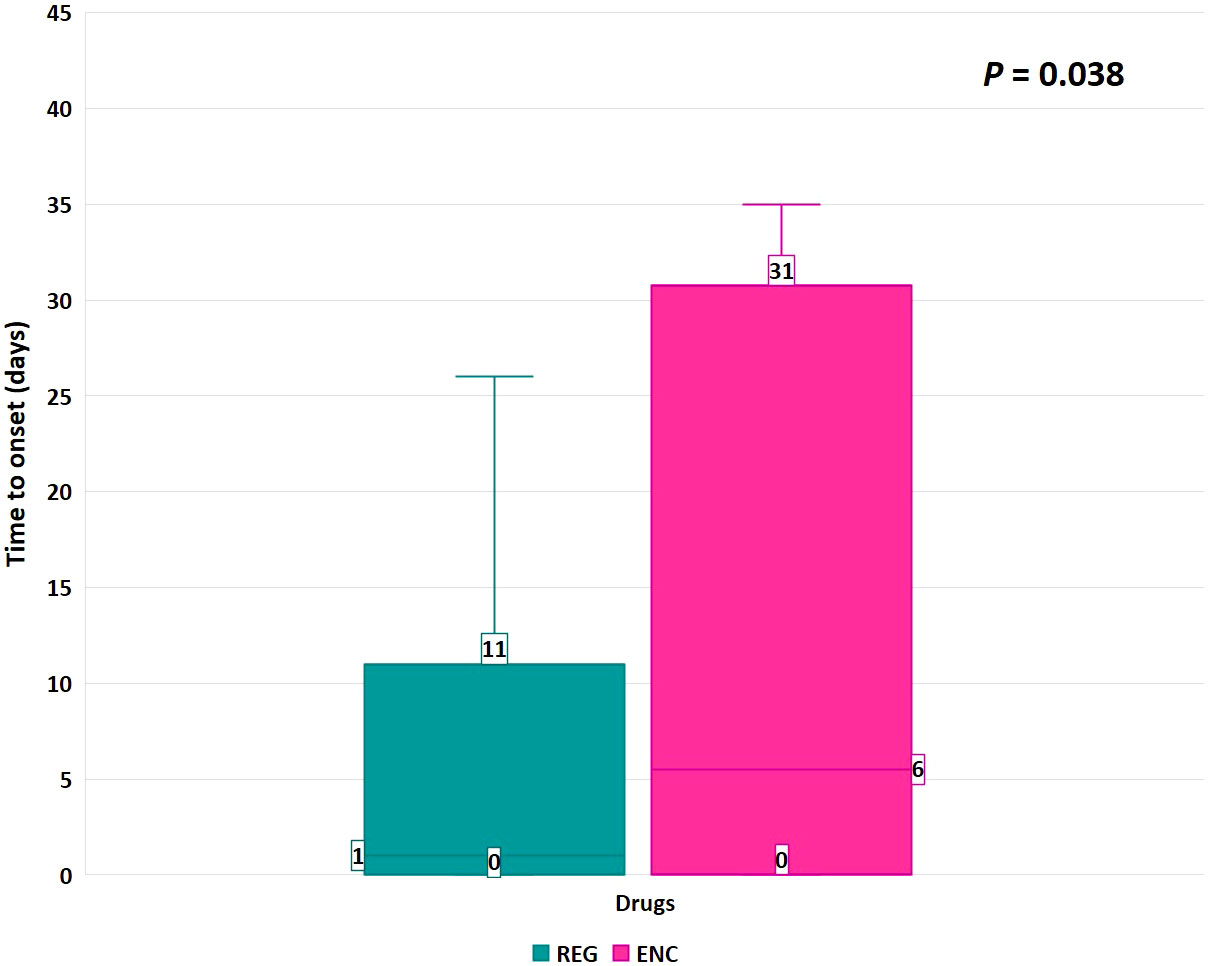
Time to onset of neuropsychiatric ADRs. Data are reported as box plot with the box drawing from Q1 to Q3 and the horizontal line drawing in the middle to denote the median. REG, regorafenib; ENC, encorafenib.

The median (Q1-Q3) TTO of neuropsychiatric ADRs was lower with REG [1 (0–11) day] than with ENC [6 (0–31) days] (*p* = 0.038) (**Figure 2**). Considering that each report could contain one ADR belonging to the SOC “Nervous system disorders” and one belonging to “Psychiatric disorders”, nervous system disorders accounted for 1,194 reports (88.0%), while psychiatric disorders accounted for 367 cases (27.0%). Neuropathy peripheral (*n* = 273; 20.1%), headache (*n* = 263; 19.4%), dizziness (*n* = 129; 9.5%), paraesthesia (*n* = 103; 7.6%), and confusional state (*n* = 103; 7.6%) were the most frequently reported neuropsychiatric ADRs by PT, especially for REG, except for headache, which was mainly related to ENC (*n* = 17; 23.6%).

### Disproportionality analysis

Neuropsychiatric ADRs with a disproportionality signal primarily corresponded to ADRs already reported in the FDA labels at the time of the study. These included headache for both ENC and REG, dizziness, tremor, lethargy, all kind of encephalopathies, confusional state, eating disorders, and mental status changes, which were specific to REG. Neuropathy peripheral, insomnia, and polyneuropathy were specific to ENC. However, new potential safety signals have been identified. For REG, the main unexpected disproportions, identified using consistent frequentist ROR and Bayesian IC, belonging to nervous system disorders were thermohyperaesthesia (*n* = 3; ROR = 578.67, CI 95% = 176.54-1,896.81; IC = 1.93, IC_025_-IC_075 _= 0.75-3.12), neuropathy peripheral (*n* = 265; ROR = 19.48, CI 95% = 17.52-22.47; IC = 2.89, IC_025_-IC_075 _= 2.77-3.02), hypogeusia (*n* = 5; ROR = 17.87, CI 95% = 7.42-43.01; IC = 1.95, IC_025_-IC_075 _= 1.07-2.83), hyperaesthesia (*n* = 18; ROR = 12.56, CI 95% = 7.90-19.96; IC = 2.25, IC_025_-IC_075 _= 1.79-2.72), slow speech (*n* = 3; ROR = 10.40, CI 95% = 3.35-32.29; IC = 1.49, IC_025_-IC_075 _= 0.36-2.62), taste disorder (*n* = 41; ROR = 9.91, CI 95% = 7.29-13.49; IC = 2.18, IC_025_-IC_075 _= 1.88-2.49), hypersomnia (*n* = 37; ROR = 9.46, CI 95% = 6.85-13.08; IC = 2.13, IC_025_-IC_075 _= 1.81-2.46), incoherent (*n* = 8; ROR = 7.97, CI 95% = 3.98-15.96; IC = 1.73, IC_025_-IC_075 _= 1.04-2.42), poor quality sleep (*n* = 18; ROR = 6.56, CI 95% = 4.13-10.42; IC = 1.74, IC_025_-IC_075 _= 1.27-2.20), burning sensation (*n* = 63; ROR = 6.12, CI 95% = 4.77-7.85; IC = 1.76, IC_025_-IC_075 _= 1.51-2.01), altered state of consciousness (*n* = 15; ROR = 5.50, CI 95% = 3.31-9.14; IC = 1.57, IC_025_-IC_075 _= 1.06-2.07), neuralgia (*n* = 17; ROR = 5.10, CI 95% = 3.17-8.21; IC = 1.51, IC_025_-IC_075 _= 1.04-1.99), dysstasia (*n* = 21; ROR = 5.05, CI 95% = 3.29-7.75; IC = 1.53, IC_025_-IC_075 _= 1.10-1.95), peripheral sensory neuropathy (*n* = 4; ROR = 5.02, CI 95% = 1.88-13.38; IC = 1.24, IC_025_-IC_075 _= 0.26-2.22), sciatica (*n* = 9; ROR = 4.70, CI 95% = 2.45-9.05; IC = 1.37, IC_025_-IC_075 _= 0.71-2.02), hypoaesthesia (*n* = 102; ROR = 4.52, CI 95% = 3.71-5.50; IC = 1.47, IC_025_-IC_075 _= 1.28-1.67), and motor dysfunction (*n* = 6; ROR = 4.31, CI 95% = 1.94-9.61; IC = 1.23, IC_025_-IC_075 _= 0.43-2.03). For psychiatric disorders with REG, an unknown significant disproportionality was shown for disorientation (*n* = 26; ROR = 4.00, CI 95% = 2.72-5.88; IC = 1.33, IC_025_-IC_075 _= 0.94-1.71), delirium (*n* = 20; ROR = 3.68, CI 95% = 2.37-5.71; IC = 1.24, IC_025_-IC_075 _= 0.80-1.68), depressed mood (*n* = 13; ROR = 1.85, CI 95% = 1.07-3.19; IC = 0.58, IC_025_-IC_075 _= 0.04-1.13), and insomnia (*n* = 63; ROR = 1.48, CI 95% = 1.15-1.89; IC = 0.38, IC_025_-IC_075 _= 0.13-0.63).

For ENC, consistent disproportionalities of neuropsychiatric ADRs not reported in the FDA label were observed. These included brain oedema (*n* = 3; ROR =14.11, CI 95% =4.53-43.93; IC = 2.64, IC_025_-IC_075 _= 1.50-3.78) and cognitive disorders (*n* = 3; ROR = 4.71, CI 95% = 1.51-14.66; IC = 1.54, IC_025_-IC_075 _= 0.41-2.68) for nervous system disorders, and depressed mood (*n* = 4; ROR = 5.75, CI 95% = 2.15-15.39; IC = 1.74, IC_025_-IC_075 _= 0.76-2.73) for psychiatric disorders (**Table 2**).

**Table 2:** Disproportionality analyses, included ROR and IC, and notoriety based on FDA label for neuropsychiatric ADRs related to ENC and REG.

System Organ Class	Preferred Term	ENC				REG				Total
		N	ROR (95% CI)	IC (IC_025_-IC_075_)	Unexpected	N	ROR (95% CI)	IC (IC_025_-IC_075_)	Unexpected	
Nervous system disorders	Neuropathy Peripheral	8	**5.74 (2.85-11.56)**	1.73 (1.03-2.43)	No	265	**19.84 (17.52-22.47)**	2.89 (2.77-3.02)	Yes	273
	Headache	17	**1.74 (1.07-2.82)**	0.54 (0.05-1.02)	No	246	**2.55 (2.24-2.90)**	0.90 (0.77-1.03)	No	263
	Dizziness	4	0.49 (0.18-1.31)			125	**1.55 (1.30-1.85)**	0.43 (0.25-0.61)	No	129
	Hypoaesthesia	1	NA			102	**4.52 (3.71-5.50)**	1.47 (1.28-1.67)	Yes	103
	Paraesthesia					82	**3.02 (2.43-3.76)**	1.08 (0.86-1.30)	Yes	82
	Burning Sensation					63	**6.12 (4.77-7.85)**	1.76 (1.51-2.01)	Yes	63
	Somnolence	3	1.07 (0.34-3.33)			50	**1.78 (1.35-2.36)**	0.57 (0.29-0.85)	Yes	53
	Tremor	1	NA			43	**1.53 (1.14-2.07)**	0.42 (0.12-0.72)	No	44
	Taste Disorder	2	NA			41	**9.91 (7.29-13.49)**	2.18 (1.88-2.49)	Yes	43
	Balance Disorder					38	**3.04 (2.21-4.18)**	1.08 (0.76-1.40)	Yes	38
	Hypersomnia					37	**9.46 (6.85-13.08)**	2.13 (1.81-2.46)	Yes	37
	Lethargy	1	NA			26	**2.92 (1.98-4.29)**	1.03 (0.65-1.42)	No	27
	Hepatic Encephalopathy					25	**16.28 (10.98-24.13)**	2.52 (2.13-2.92)	No	25
	Loss Of Consciousness	1	NA			24	1.25 (0.83-1.86)			25
	Memory Impairment	3	1.56 (0.50-4.86)			20	1.03 (0.67-1.61)			23
	Speech Disorder	2	NA			21	**2.35 (1.53-3.61)**	0.82 (0.39-1.25)	Yes	23
	Cerebrovascular Accident	4	1.48 (0.55-3.97)			18	0.66 (0.42-1.05)			22
	Dysstasia	1	NA			21	**5.05 (3.29-7.75)**	1.53 (1.10-1.95)	Yes	22
	Seizure	1	NA			19	0.62 (0.39-0.9)			20
	Amnesia	1	NA			18	1.53 (0.96-2.43)			19
	Hyperaesthesia					18	**12.56 (7.90-19.96)**	2.25 (1.79-2.72)	Yes	18
	Neuralgia	1	NA			17	**5.10 (3.17-8.21)**	1.51 (1.04-1.99)	Yes	18
	Poor Quality Sleep					18	**6.56 (4.13-10.42)**	1.74 (1.27-2.20)	Yes	18
	Depressed Level of Consciousness					17	**2.59 (1.61-4.17)**	0.90 (0.43-1.38)	Yes	17
	Syncope	3	1.63 (0.52-5.07)			14	0.76 (0.45-1.28)			17
	Altered State of Consciousness					15	**5.50 (3.31-9.14)**	1.57 (1.06-2.07)	Yes	15
	Cerebral Infarction					13	**3.25 (1.89-5.61)**	1.10 (0.55-1.64)	Yes	13
	Cognitive Disorder	3	**4.71 (1.51-14.66)**	1.54 (0.41-2.68)	Yes	10	1.56 (0.84-2.90)			13
	Encephalopathy					13	**3.27 (1.89-5.63)**	1.10 (0.56-1.65)	No	13
	Posterior Reversible Encephalopathy Syndrome					12	**9.10 (5.16-16.05)**	1.93 (1.36-2.49)	No	12
	Dysgeusia					11	1.02 (0.56-1.85)			11
	Ageusia					10	**2.51 (1.35-4.68)**	0.85 (0.23-1.47)	Yes	10
	Cerebral Haemorrhage					10	**1.93 (1.04-3.59)**	0.61 (-0.01-1.23)	No	10
	Sciatica	1	NA			9	**4.70 (2.45-9.05)**	1.37 (0.71-2.02)	Yes	10
	Coma					9	0.91 (0.47-1.74)			9
	Coma Hepatic					9	**64.46 (33.40-124.42)**	2.70 (2.04-3.35)	No	9
	Disturbance In Attention	1	NA			8	0.99 (0.49-1.98)			9
	Dysarthria					9	1.51 (0.78-2.90)			9
	Mental Impairment	2	NA			7	1.80 (0.86-3.79)			9
	Brain Oedema	3	**14.11 (4.53-43.93)**	2.64 (1.50-3.78)	Yes	5	2.33 (0.97-5.60)			8
	Incoherent					8	**7.97 (3.98-15.96)**	1.73 (1.04-2.42)	Yes	8
	Movement Disorder					8	1.60 (0.80-3.20)			8
	Polyneuropathy	5	**31.22 (12.93-75.39)**	3.43 (2.55-4.31)	No	3	1.85 (0.60-5.73)			8
	Unresponsive To Stimuli					8	**2.24 (1.12-4.48)**	0.74 (0.04-1.43)	Yes	8
	Epilepsy					7	1.70 (0.81-3.58)			7
	Hypogeusia	2	NA			5	**17.87 (7.42-43.01)**	1.95 (1.07-2.83)	Yes	7
	Migraine	1	NA			6	0.45 (0.20-1.00)			7
	Motor Dysfunction	1	NA			6	**4.31 (1.94-9.61)**	1.23 (0.43-2.03)	Yes	7
	Central Nervous System Lesion					6	**2.87 (1.29-6.40)**	0.92 (0.12-1.72)	Yes	6
	Hemiparesis					6	2.14 (0.96-4.76)			6
	Transient Ischaemic Attack					6	1.15 (0.51-2.55)			6
	Aphasia					5	1.03 (0.43-2.48)			5
	Head Discomfort					5	2.15 (0.89-5.16)			5
	Sensory Disturbance					5	1.98 (0.82-4.76)			5
	Cerebral Ischaemia					4	**3.30 (1.24-8.81)**	0.97 (-0.01-1.95)	Yes	4
	Facial Paralysis	1	NA			3	1.44 (0.47-4.48)			4
	Ischaemic Stroke					4	1.57 (0.59-4.19)			4
	Peripheral Sensory Neuropathy					4	**5.02 (1.88-13.38)**	1.24 (0.26-2.22)	Yes	4
	Presyncope					4	1.23 (0.46-3.29)			4
	Restless Legs Syndrome					4	1.54 (0.58-4.12)			4
	Coordination Abnormal					3	0.70 (0.23-2.17)			3
	Haemorrhagic Stroke					3	1.40 (0.45-4.34)			3
	Nervous System Disorder					3	0.55 (0.18-1.70)			3
	Paralysis					3	1.04 (0.33-3.22)			3
	Parosmia					3	2.01 (0.65-6.23)			3
	Slow Speech					3	**10.40 (3.35-32.29)**	1.49 (0.36-2.62)	Yes	3
	Thermohyperaesthesia					3	**578.67 (176.54-1,896.81)**	1.93 (0.75-3.12)	Yes	3
Psychiatric disorders	Confusional State	2	NA			101	**3.62 (2.97-4.41)**	1.26 (1.06-1.45)	No	103
	Insomnia	10	2.37 (1.27-4.44)		No	63	**1.48 (1.15-1.89)**	0.38 (0.13-0.63)	Yes	73
	Anxiety	1	NA			33	0.73 (0.52-1.03)			34
	Eating Disorder	1	NA			28	**9.33 (6.43-13.53)**	2.09 (1.72-2.46)	No	29
	Disorientation					26	**4.00 (2.72-5.88)**	1.33 (0.94-1.71)	Yes	26
	Depression	1	NA			23	0.59 (0.39-0.90)			24
	Delirium					20	**3.68 (2.37-5.71)**	1.24 (0.80-1.68)	Yes	20
	Depressed Mood	4	**5.75 (2.15-15.39)**	1.74 (0.76-2.73)	Yes	13	**1.85 (1.07-3.19)**	0.58 (0.04-1.13)	Yes	17
	Hallucination					13	1.05 (0.61-1.81)			13
	Mental Status Changes					10	**2.41 (1.29-4.47)**	0.81 (0.19-1.43)	No	10
	Sleep Disorder	1	NA			9	0.98 (0.51-1.88)			10
	Restlessness					7	1.28 (0.61-2.68)			7
	Thinking Abnormal					7	1.57 (0.75-3.29)			7
	Irritability	1	NA			5	0.54 (0.22-1.30)			6
	Middle Insomnia					5	2.05 (0.85-4.29)			5
	Stress	1	NA			4	0.41 (0.16-1.10)			5
	Abnormal Behaviour					4	0.65 (0.24-1.74)			4
	Agitation					4	0.28 (0.11-0.76)			4
	Fear					4	0.92 (0.34-2.44)			4
	Hallucination, Visual					4	1.41 (0.53-3.76)			4
	Nervousness					4	0.34 (0.13-0.92)			4
	Suicidal Ideation					4	0.30 (0.11-0.80)			4
	Aggression					3	0.38 (0.12-1.17)			3
	Delusion					3	1.22 (0.39-3.79)			3
	Frustration Tolerance Decreased					3	1.88 (0.61-5.84)			3
	Mental Disorder					3	0.48 (0.15-1.48)			3
	Panic Attack					3	0.57 (0.18-1.76)			3
	Personality Change					3	2.00 (0.65-6.21)			3

Significant RORs are in bold type.

NA, not applicable because there were fewer than three reports.

CI, confidence interval; ENC, encorafenib; IC, information component; REG, regorafenib; ROR, Reporting Odds Ratio.

## Discussion

This study focused on the neuropsychiatric disorders associated with REG and ENC approved for the treatment of mCRC and reported in the FAERS database. The decision to evaluate this safety concern was motivated by the lack of SRS database studies that aimed to detect ADRs for TKIs in mCRC.

The distribution of reports over the years has been influenced by the timing of approval and clinical use of REG and ENC. Neuropsychiatric ADRs have predominantly been reported for REG. However, the recent increase in their reporting compared to all other reports, especially in 2021, may be attributed to the recent approval of ENC in combination with CET for mCRC (12). The frequency of neuropsychiatric ADRs with REG appeared to be higher than what was observed in a previous study that focused on neuropsychiatric ADRs with oral TKIs approved for gastrointestinal stromal tumor (GIST) and was based on the EudraVigilance database (28.6% vs. 13.0%) (16). This difference could be attributed to the higher incidence of CRC compared to GIST in the entire population, as well as the use of different SRS databases for the analysis. Looking at the characteristics of reports, neuropsychiatric ADRs were almost equally distributed by gender; however, they were mainly reported for adults than all other reports, especially for patients aged between 50 and 64 years. This could be explained by the rising trend of CRC diagnosis in young adults, which is facilitated by recommended screening methods enabling early detection of CRC (23). Nevertheless, a previous study reported that mental and cognitive disorders are more common in elderly patients diagnosed with CRC (24). The literature data on gender and neuropsychiatric ADRs are controversial. Some studies have reported that females with CRC are more prone to develop toxicity associated with targeted therapies than males (25, 26). However, serious neurological symptoms and mood disorders were mainly observed in men rather than women treated with target therapies (27). Additionally, neuropsychiatric ADRs were primarily reported by consumers, as stated in a previous study (28). Although FAERS is a global database, a large portion of spontaneous reports, including those related to neuropsychiatric ADRs, originated from America. This could potentially be attributed to the growing manufacturing activities and investments made by pharmaceutical companies leading to an increased request for efficient pharmacovigilance service (29).

Considering the seriousness and outcomes, a higher percentage of serious ADRs was shown for neuropsychiatric reports compared to all other reports, particularly those with disabled outcomes. In a pharmacovigilance study, serious neurological ADRs were mainly reported for ENC in combination with binimetinib in cases where melanoma was the indicated condition (17). Furthermore, REG seemed to have a higher likelihood of developing serious ADRs, including neuropsychiatric ones, in patients with mCRC as well as in subjects with GIST (16). This could potentially be explained by the invasive nature of cancer, but also by the lower survival benefit resulting from the oral TKI discontinuation after the occurrence of serious ADRs (5, 30).

The median TTO of neuropsychiatric ADRs was lower with REG compared to ENC. Based on the literature, the median TTO of ADRs related to the nervous system disorders was 225 days with ENC+CET (31) while it was 54 days for peripheral neuropathy with ENC+binimetinib (17). Another study investigating all neuropsychiatric ADRs associated with TKIs in GIST patients showed a median TTO of 22 days for REG (16). These findings were higher than it was observed in the present study, likely due to limited cases reporting TTO, the involvement of different types of cancer, or the presence of comorbidities.

Neuropsychiatric ADRs most commonly reported for REG included neuropathy peripheral, headache, dizziness, paraesthesia, and confusional state, as observed in several studies (10, 16, 32, 33). Headache was also frequently reported for ENC, with approximately 20% of patients receiving ENC+CET experiencing this ADR, as indicated in the literature (5, 31). Additionally, the FDA label for REG highlights headache as very common, without specifically mentioning of other neuropsychiatric ADRs. However, the European Medicines Agency (EMA) Summary of Product Characteristics (SmPC) for REG mentions tremor, peripheral neuropathy, and posterior encephalopathy syndrome (PRES) (34).

The disproportional analysis revealed some potential unexpected signals that were not reported in the FDA labels, with certain mechanisms yet to be defined. For REG, a statistically significant ROR was found for nerve disorders and associated symptoms, such as neuropathy peripheral, peripheral sensory neuropathy, sciatica, neuralgia, hyperaesthesia, thermohyperaesthesia, hypoaesthesia, and burning sensation. Although REG is related to low direct neurotoxicity, it could have potential side effects targeting VEGF, including vascular toxicity that could lead to neurotoxicity as a consequence (35). The onset of nerve disorders may occur as a result of unexpected stimulation of the MAPK pathway, leading to increased growth of Schwann cells. Conversely, selective activation of the MAPK signaling pathway or, alternatively, overexpression of RAF showed a negative impact on Schwann cell differentiation (36, 37). Moreover, aberrant immune activation against peripheral nerves could lead to treatment-induced inflammatory demyelinating peripheral neuropathy, particularly due the significant role played by the MAPK pathway in the production of proinflammatory cytokines (38, 39). However, a potential correlation with pre-existing nervous or psychiatric comorbidities, as well as the concomitant use of other drugs cannot be excluded. Also, prior treatment lines, including cytotoxic drugs that may cause neurotoxicity, such as oxaliplatin, should be taken into account when reporting neuropsychiatric ADRs in pretreated CRC patients (40). Furthermore, the role of prior radiation therapy and the administration of immune checkpoint inhibitors (ICIs) could impair neuronal repair and exacerbate nerve toxicity with REG (41, 42). Moreover, hyperaesthesia, thermohyperaesthesia, hypoaesthesia, and burning sensation, can be considered as consequential symptoms of neuropathy, as already known in the literature (18).

A disproportionality analysis revealed a significant association between REG and taste disorders, including hypogeusia. Disturbed taste is a well-known ADR of REG and mentioned in the EMA SmPC but not in the FDA Prescribing Information. In an exploratory study, taste disorders were reported in 55% of patients treated with REG and were found to be associated with a lower QoL (43). The onset of taste disorders, such as hypogeusia, may be linked to an uncharacterized neuropathic etiology, possibly attributed to the involvement of the chorda tympani nerve responsible for taste and salivary innervation (44). Another significant and unexpected disproportion was observed in relation to slow speech. Speech disorders may be associated with the development of stomatitis, which is caused by DNA damage to the mucosal surface (45); otherwise, a case report described a patient undergoing treatment with REG who experienced slurred speech, probably due to the presence of brain metastases (46). Focusing on motor dysfunctions, such as dysstasia, several case reports (46–48) have suggested a potential cause: the interference of TKIs with cerebral signal pathway or distress resulting from their antiangiogenic action, which may lead to brain-related complications in patients with predisposing conditions. Moreover, a genetic polymorphism in pharmacokinetic and pharmacodynamic pathways could also contribute to these ADRs (46).

Regarding sleep disturbances, there was a significant unknown disproportionality for poor-quality sleep, hypersomnia and, insomnia within the SOC psychiatric disorder, for REG. TKIs are commonly associated with sleep disorders, as evidenced by a clinical study where 14% of TKI-treated patients had serious sleep disturbances, with improvements noted upon TKI discontinuation (49). Furthermore, the onset of sleep disturbance is frequently reported as a troublesome symptom in patients undergoing REG treatment (50). However, the onset of sleep disorders may be related to the pathogenesis and progression of CRC itself; in fact, more than 70% of CRC patients have reported sleeping issues associated with circadian disruption. The dysregulation of circadian genes in cancer leads to the downregulation of PER2 and the upregulation of β-catenin protein levels, which can cause the proliferation of CRC cells (51). Additionally, the development of insomnia may not be specific to the type of drug used; a previous study found no differences among chemotherapy, ICIs, and target therapy (52–54).

The consistent disproportionality of altered state of consciousness, incoherent status, disorientation, and delirium is not reported in the FDA label for REG. Similarly, cognitive disorders are not mentioned in the FDA label for ENC. Nevertheless, altered mental status is reported in the EMA SmPC for REG (34). Cognitive impairment can be considered as an effect resulting from the activation of multiple signaling pathways, including RAF/MAPK and VEGF/VEGFR2/mTOR, which subsequently affect various cellular processes associated with corticogenesis (33, 41, 55). Cognitive disorders have been also found to be three to five times higher in CRC patients compared to healthy control, with higher rates of impairment observed in women than men (56).

Moreover, depressed mood was identified as a potential unknown disproportionality signal for both REG and ENC. Depressed mood may be associated with a diagnosis of depression following the diagnosis of CRC, whether or not it is related to the administration of BRAFi (57, 58). The onset of depressed mood with ENC may be linked to its concomitant use with CET, as mentioned in CET FDA label (59). Depression could also be influenced by the impact on QoL of typical CET toxicities, for instance diarrhea and skin rash. It’s worth noting that CET-related skin rash occurs much less frequently and is less severe when combined with ENC compared to monotherapy (60). However, a possible correlation with ENC cannot be excluded.

Considering brain oedema, it is an unknown effect associated with ENC that could be also caused by brain metastases rather than ENC. Localized or peripheral oedema was reported in the EMA SmPC (61), but not in the FDA label. Oedema was frequently observed with BRAFi/MEKi combinations (62), but no literature evidence was found for the association with CET.

### Strengths and limitations

The SRS analyses are the most common pharmacovigilance methods and the best ones to generate potential signals that require further validation (63). The FAERS database allows for the detection of neuropsychiatric ADRs related to the use of oral TKIs approved for mCRC, including rare and serious ones, due to the extensive collection of reports. No previous studies aimed to evaluate REG- and ENC-related neuropsychiatric ADRs in mCRC. The increased use oral TKIs as second line therapy and the recent approval of the association ENC+CET, make further safety investigations on these drugs necessary, especially for neuropsychiatric ADRs. Therefore, the major strength of this study is that it contributed to the cumulative knowledge about the safety profile of oral TKIs using a global database and merging a disproportionality approach with case/non-case evaluation (21). Patients diagnosed with mCRC have worse health related QoL, which can be influenced by the use of oral TKIs as second line therapy following previous treatments, including chemotherapy. Negative effects can also arise from the duration of the tumor course and the progression of metastases. Indeed, it would be interesting to analyze, in a real-world setting, whether the patterns of metastatic disease in CRC (e.g., bone or brain metastases) could influence the development of neuropsychiatric ADRs. Additionally, pre-existing nervous or psychiatric comorbidities, or those consequent to the cancer diagnosis, such as depression, should be considered. For all the aforementioned reasons, avoiding the onset of neuropsychiatric ADRs, that could further worsen QoL with conditions, such as cognitive impairment and nerve disorders, could be useful in improving the management and compliance of patients with cancer, including CRC, as observed in previous studies (16, 64, 65).

However, global databases, including FAERS, suffer from several limitations that should be taken into account when analyzing ADRs. These limitations include underreporting and overreporting phenomena of certain ADRs. The lack of a denominator which represents the total number of patients exposed to a specific product, also poses a challenge. Moreover, due to the recent approval of ENC in comparison to REG, fewer than 10% of neuropsychiatric ADRs had ENC as primary suspect. The background reporting rate can also be influenced by the association between an ADR and the use of secondary suspect drugs, including CET in ENC-related reports, making it difficult to establish a causal relationship and reducing the sensitivity of the analysis. Additionally, important information such as demographic characteristics, TTO, dechallenge, rechallenge, comorbidities, and comedications maybe be missing from the reports (66–70).

Furthermore, oral TKIs are prescribed for advanced and mCRC, which means that some serious neuropsychiatric ADRs may be attributed to neoplasm progression, delayed ADRs, or comorbidities in mCRC patients treated with oral TKIs (17, 71). Despite these limitations, reports obtained from the FAERS database help in better characterizing the safety profile of ENC and REG, which is particularly important for preventing neuropsychiatric ADRs in mCRC patients and assisting oncologists in effectively managing potential complications, thus improving patient’s health related QoL.

## Conclusions

This study confirms the crucial role of the FAERS database in evaluating neuropsychiatric ADRs related to oral TKIs approved for mCRC. REG has higher probability of reporting neuropsychiatric ADRs compared to ENC, which could be attributed to its previous approval for mCRC. The case/non-case analysis and the calculation of ROR have highlighted some ADRs that have not been extensively reported in the literature but are worth discussing, such as nerve disorders, taste disorders, slow speech, and sleep disturbances for REG, and cognitive disorders, brain oedema, and depressed mood for ENC.

Neuropsychiatric ADRs can significantly impact a patient’s QoL and treatment outcomes. By considering pre-existing nervous or psychiatric comorbidities, oncologists can better assess the potential risks and take appropriate measures to minimize the occurrence of ADRs or manage them effectively. However, further real-world studies are necessary to achieve a better understanding of these ADRs and their impact on patients’well-being.

## Data availability statement

The datasets presented in this study can be found in online repositories. The names of the repository/repositories and accession number(s) can be found below: https://fis.fda.gov/extensions/FPD-QDE-FAERS/FPD-QDE-FAERS.html.

## Ethics statement

The study was approved by the Ethics Committee of University Hospital of Messina (n. 40/23 of 28th February 2023). Written informed consent from the participants was not required to participate in this study in accordance with the national legislation and the institutional requirements.

## Author contributions

MAB: Conceptualization, Formal Analysis, Methodology, Visualization, Writing – original draft, Writing – review & editing. GR: Conceptualization, Formal Analysis, Methodology, Visualization, Writing – original draft. EES: Formal Analysis, Visualization, Writing – original draft. GC: Visualization, Writing – original draft. TF: Visualization, Writing – review & editing. MS: Visualization, Writing – review & editing. DS: Visualization, Writing – review & editing. ES: Supervision, Validation, Visualization, Writing – review & editing. NS: Supervision, Validation, Visualization, Writing – review & editing.
